# Extracellular vesicle analytical science loses a touch of creativity and kindness

**DOI:** 10.1002/jev2.12504

**Published:** 2024-09-10

**Authors:** Paolo Bergese, Marcella Chiari, Alessandro Gori, Benedetta Bussolati, Pietro Parisse, the EVIta Board

**Affiliations:** ^1^ Department of Molecular and Translational Medicine University of Brescia Brescia Italy; ^2^ Institute of Chemical and Technological Science “Giulio Natta” National Research Council Milan Italy; ^3^ Department of Medical Sciences University of Torino Torino Italy; ^4^ Institute of Materials, Department of Physical Sciences and Technologies of Matter National Research Council (CNR) Trieste Italy


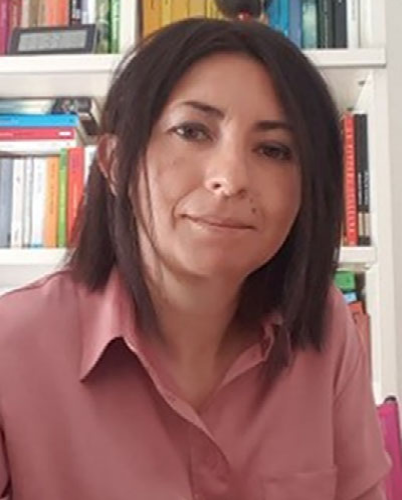


It is with profound sadness and sorrow that we announce the unexpected passing of our cherished friend and colleague, Marina Cretich. Marina, a renowned scientist in the field of bioanalytical chemistry, passed away on June 29th 2024, leaving behind a heritage of invaluable contributions to science. Her career was marked by a relentless pursuit of knowledge and an unwavering dedication to advancing scientific understanding.

Marina graduated in Biological Sciences with a specialization in Molecular Biology from the University of Milano in 1998 with Prof. Piergiorgio Righetti. She then joined the National Research Council of Italy (CNR), where she served as a Researcher at the Institute of Chemistry for Molecular Recognition in Milan, becoming a key figure in the Analytical Microsystem laboratory guided by Dr. Marcella Chiari. Thanks to the interdisciplinarity of her approach, she made significant strides in the progress of advanced methods and materials for bio‐molecular recognition, together with high‐sensitive and selective biosensors, providing new tools for detecting biomolecules with unprecedented accuracy and efficiency. Her strong focus on integrating bioanalytical techniques, microfluidics and detection tools, enabled more rapid and precise analyses, enhancing the capabilities of lab‐on‐a‐chip devices, towards point‐of‐care diagnostics.

These studies paved the road to her approach to extracellular vesicles, addressing the challenges posed by their separation and analysis. Specifically, Marina was working on affinity‐capturing protocols from complex samples for EV isolation and on microarray platforms for their molecular characterization (Daaboul et al., 2016). Her interest in the field was raised by participating in the EU project INDEX, coordinated by Dr Marcella Chiari, and became true love for this fascinating and challenging area. This led Marina to establish her own research team and to find the Extracellular Vesicle Lab at SCITEC‐CNR. Soon after, she was the Coordinator of the EU project MARVEL, a multi‐partner project at the intersection of chemistry and technology, biology and translational medicine, which was centred around the use of membrane‐sensing peptides (MSP) as enabling tools for the multiscale EV isolation (Gori et al., 2020, Gori et al, 2024). She was a true pioneer in this field and she consolidated a leading expertise in the area of ultrasensitive EV analysis, aiming to fill existing gaps in the clinical translation of EVs in diagnostics. Her contribution is witnessed by remarkable scientific contributions (Frigerio et al., 2022; Musicò et al., 2024) and, despite very recent, the concepts and technologies that she developed set the basis for an ever‐increasing number of preclinical and clinical collaborations encompassing EV analysis in the fields of neurodegeneration, cancer, and heart diseases.

In recent years, she also dedicated herself to the translation of the analytical equipment she developed to support new explorations of the nanostructured secretome landscape. For example, she participated in opening the path to the study of the EV biomolecular corona, which is determined by the interaction between EVs with the repertoire of biomolecules and other extracellular nanoparticles (e.g., lipoproteins) populating biological fluids, and has been shown to be crucial in determining key physicochemical properties and functions of EVs (Musicò et al., 2024; Ridolfi et al., 2023).

The international positioning achieved by Marina in the field of EVs is also witnessed by numerous invited seminars, oral communications and activities conducted within the framework of the ISEV community. It is worth highlighting her lectures at the Education Day of the annual ISEV meeting in 2022 on Single Vesicle Analysis techniques, and in 2024 during the Workshop “Tiny Packages Big Impact” at the University of Virginia, Charlottesville. She was also Co‐Chair of the ISEV workshop “QuantitatEVs (J. Extracell. Biol. 2024;3:e137”) and contributing author of the “Minimal information for studies of extracellular vesicles (MISEV2023): From basic to advanced approaches”. At the national level, she was amongst the founders of the Italian Society for Extracellular Vesicles (EVIta), and elected member of the EVIta Board since 2022. She was also a promoter of a multi‐department initiative within the National Research Council of Italy (CNR‐EV‐NET: Joining forces in the study of Extracellular Vesicles).

Beyond her scientific achievements, Marina was a beloved mentor and colleague. She dedicated herself to students, nurturing their talents and guiding their research endeavours. Her commitment to education and her ability to inspire young scientists will be remembered as one of her most significant legacies.

Marina's passing is a profound loss to the scientific community because of her brilliance, dedication, and profound love for the pursuit of knowledge. However, she will be remembered not only for her scientific contributions, but also for her kindness, generosity, reliability, and the positive impact she had on all who had the privilege of knowing her. Marina exemplified dedication and professionalism, serving as a beacon of inspiration and a cornerstone for us all.
